# Sex Differences in the Renal Function Decline of Patients with Type 2 Diabetes

**DOI:** 10.1155/2016/4626382

**Published:** 2016-05-09

**Authors:** Ayami Kajiwara, Ayana Kita, Junji Saruwatari, Hiroko Miyazaki, Yuki Kawata, Kazunori Morita, Kentaro Oniki, Akira Yoshida, Hideaki Jinnouchi, Kazuko Nakagawa

**Affiliations:** ^1^Division of Pharmacology and Therapeutics, Graduate School of Pharmaceutical Sciences, Kumamoto University, 5-1 Oe-honmachi, Chuo-ku, Kumamoto 862-0973, Japan; ^2^Center for Clinical Pharmaceutical Sciences, Kumamoto University, 5-1 Oe-honmachi, Chuo-ku, Kumamoto 862-0973, Japan; ^3^Jinnouchi Clinic, Diabetes Care Center, 6-2-3 Kuhonji, Chuo-ku, Kumamoto 862-0976, Japan

## Abstract

*Aims*. We aimed to investigate the sex differences in the renal function decline among patients with type 2 diabetic mellitus (T2DM), focusing on the differences in the risk factors at early stage of renal dysfunction.* Methods*. A clinic-based retrospective longitudinal study (follow-up duration: 8.1 ± 1.4 years) was conducted to assess the sex differences in the annual estimated glomerular filtration rate (eGFR) change in 344 (247 male and 97 female) Japanese T2DM patients. The sex differences in the risk factors of annual eGFR decline were subjected to linear regression analyses.* Results*. The mean annual eGFR change was −3.5 ± 2.7%/year in females and −2.0 ± 2.2%/year in males (*P* < 0.001). Baseline retinopathy and proteinuria were significantly associated with a larger eGFR decline, irrespective of sex, while HbA1c and LDL-cholesterol levels were significantly associated with an eGFR decline in females only. Interactive effects were observed between sex and the HbA1c, LDL-cholesterol, retinopathy, or proteinuria levels on the annual eGFR decline.* Conclusions*. The increased susceptibility to poor metabolic control seemed to contribute to a higher risk of renal dysfunction in females with T2DM. Our study highlights the importance of aggressive therapeutic intervention to improve metabolic profiles at early stage, especially in females.

## 1. Introduction

Elucidating sex differences in diabetes is a necessary step toward personalized medicine and improved public health [1–3]. There is growing evidence to indicate that diabetic females have a greater relative risk of cardiovascular disease (CVD) than diabetic males [4–9]. The Japan Public Health Centre-based prospective study revealed that diabetes was associated with an increased risk of death by CVD: hazard ratio (HR) 1.76 [95% confidence interval (CI), 1.53–2.02] for males, and HR 2.49 [95% CI, 2.06–3.01] for females [9]. Several possible explanations for the increased risk in females with diabetes were suggested, including the greater impact of CVD risk factors and/or diabetes on CVD [6, 9–12], a heavier burden of CVD risk factors due to disparities in medical treatment [4, 5, 7, 9, 10, 12] and lower achievement rates of target blood pressure values and lipid and glycemic profiles, even when they are treated similarly to males [4, 7–9, 13]. Furthermore, recent studies suggest that the increased diabetes-related risk of CVD in females is strongly associated with the chronic increase of their CVD risk profile, both in impaired glucose tolerance (IGT) and in impaired fasting glucose (IFG) states [4, 8, 10, 12, 14]. Donahue et al. reported that females who progressed from normoglycemia to a prediabetic state (fasting glucose: 100–125 mg/dL) showed greater endothelial dysfunction and a greater degree of fibrinolysis/thrombosis than males [14]. These findings may emphasize the importance of early aggressive therapeutic interventions to prevent diabetic vascular complications in females [8].

Recently, there has been increasing interest in the sex differences in the development of microvascular as well as macrovascular complications in patients with diabetes. We previously reported that Japanese females with type 2 diabetes mellitus (T2DM) exhibit a higher incidence of diabetic retinopathy (DR) (HR, 1.85; 95% CI, 1.06–3.24) and are at greater risk for progression to proliferative diabetic retinopathy (PDR) [odds ratio (OR), 2.62; 95% CI, 1.38–4.98] than males [15]. In that study, the incidence of PDR at baseline was significantly higher in females than in males, and a delay in diagnosis seemed to play a crucial role in the increased risk of DR in females [15]. On the other hand, few studies have focused on the association between sex and the development of nephropathy in T2DM patients, and it remains to be definitely determined whether a sex difference exists [10]. Several clinical reports suggest that, although the risk of development and progression of nondiabetic renal disease is lower in females than in males, such protections against renal dysfunction seem to disappear in hyperglycemic females [10, 16]. Based on the facts stated above, we hypothesized that in females with T2DM a delayed diagnosis and a poorly controlled risk profile may have a greater impact on the progression of early stage nephropathy than that which is observed in males.

On the basis of this information, the present exploratory study investigated the sex differences in longitudinal renal function among T2DM patients, with a focus on the differences in the risk factors at early stage of renal dysfunction.

## 2. Materials and Methods

### 2.1. Study Population

In this clinic-based retrospective longitudinal study, the records of 1,515 consecutive T2DM patients who visited the Jinnouchi Clinic, Diabetes Care Center in Kumamoto, Japan, between February 2002 and January 2011 were reviewed. Among them, the patients who had been treated at the clinic for less than five years, those who were over 65 years of age at baseline, and/or those with incomplete data were excluded. The study protocol was approved by the institutional ethics committees and written informed consent was obtained from each subject. The study was performed in accordance with the Declaration of Helsinki.

### 2.2. Measurements and Definitions

The patient's characteristics at their first visit (baseline) and during follow-up were obtained from their medical records. T2DM was diagnosed according to the criteria of the Japan Diabetes Society (JDS) [17] or based on the patient's history of T2DM. The value of hemoglobin A1c (HbA1c) was originally calculated using the JDS method and then converted to the internationally used HbA1c, as defined by the National Glycohemoglobin Standardization Program [17]. The body mass index (BMI) was calculated by dividing the weight in kilograms by the square of the height in meters. Overweight was defined as a BMI value of ≥25.0 kg/m^2^ [18]. Hypertension was defined as a systolic blood pressure (SBP) value of ≥140 mmHg, a diastolic blood pressure (DBP) value of ≥90 mmHg, and/or a history of hypertension [19]. Dyslipidemia was defined by the following serum concentrations: total-cholesterol, ≥5.2 mmol/L; low-density lipoprotein (LDL) cholesterol, ≥3.4 mmol/L; high-density lipoprotein (HDL) cholesterol, <1.0 mmol/L (in males) or <1.3 mmol/L (in females); triglycerides, ≥1.7 mmol/L; and/or a history of dyslipidemia [19]. DR was diagnosed by a professional ophthalmologist using direct ophthalmoscopy or fundus fluorescein angiography. DR was classified, according to the criteria determined at the third national ophthalmology conference held in 1985 [20], into the following stages: no retinopathy, nonproliferative diabetic retinopathy (NPDR), or PDR. Proteinuria was defined based on urine dipstick positivity. The estimated glomerular filtration rate (eGFR) was calculated using the Japanese eGFR-estimating equation: eGFR (mL/min/1.73 m^2^) = 194 × SCr^−1.094^  × Age^−0.287^  × 0.739 (if female). The Japanese eGFR-estimating equation has a relatively little bias across a wide range of GFR values in the Japanese population and is thus recommended for use in Japanese individuals by the Japanese Society of Nephrology [21, 22]. An eGFR decline of >4% per year was considered to be a rapid decline in eGFR [21, 23].

### 2.3. Statistical Analysis

The data are presented as the mean ± standard deviation (SD) or standard error (SE) and number (%). The continuous variables were compared by Student's *t*-test or a one-way ANOVA; the categorical variables were compared by Fisher's exact test. For each patient, a univariate linear regression model of time versus eGFR (least-squares method) was created, and the slope of the regression line was used to estimate the patient's changes in eGFR over the observational period, in accordance with the methods of a previous study [21]. The eGFR slope was expressed as percentage per year by dividing the slope by the baseline eGFR value (i.e., the annual eGFR change). Sex differences in annual eGFR decline were assessed using multivariate linear regression analysis with calculation of unstandardized partial regression coefficient (B) adjusted for age, DR, proteinuria, diabetes duration, HbA1c, SBP, LDL-cholesterol, overweight, and ever-smoking. Additionally, in order to identify the possible predictors for annual eGFR decline, we performed linear regression analyses in which the standardized partial regression coefficient (*β*) was calculated separately in males and females. The baseline characteristics including DR, proteinuria, diabetes duration, HbA1c, SBP, LDL-cholesterol, overweight, and ever-smoking were considered as the possible predictors of renal function decline. A *P* value of <0.05 in the univariate model was adopted as the entry criterion for variables to be included in the multivariable models. All *β* were adjusted for the patient's age. Furthermore, the interaction between sex and each of the factors with the annual eGFR decline was assessed using a multivariable linear model. In this model, the effects of the combination of sex and metabolic status abnormalities such as HbA1c (≥7.0%, i.e., ≥53.0 mmol/mol), LDL-cholesterol (≥3.4 mmol/L), and SBP (≥140 mmHg) on the annual eGFR decline were also assessed. The longitudinal associations between sex and these clinical parameters were analyzed using the generalized estimating equations approach. Autoregressive models were applied for the analyses with the calculations of the B and the OR and 95% CI. All of these calculations were performed using the data obtained over the entire observational period in each patient. To verify the results of the above-mentioned analyses, a supplementary analysis was performed using propensity score (PS) matching in order to reduce selection bias and confounding bias [24]. The PS was constructed using a logistic regression model. The variables included in the PS matching model are age, diabetes duration, DR, HbA1c, proteinuria, SBP, and LDL-cholesterol at baseline. Female patients were matched in a 1 : 1 ratio with male patients, using the nearest neighbor matching algorithm without replacement on the logit of the PS using a caliper of width equal to 0.001 SD of the logit of the PS. In addition, bootstrap analyses were performed to validate the findings for sex differences in renal function decline. One thousand replicated datasets were generated by random sampling with replacement and stratified according to the study population, including total study patients (i.e., unmatched) and PS-matched patients, to ensure a representative study population distribution using the individual as the sampling unit. A two-tailed *P* value of <0.05 was considered to be statistically significant. Multiple comparisons were corrected using Bonferroni's method, and *P* values of <0.05/n were considered to be statistically significant after correcting for the number of comparisons. PS matching was performed with a contributed R package (“Matching”) using the R software program (version 3.0.0, R Foundation for Statistical Computing, Vienna, Austria). Other statistical analyses were performed using the SPSS software package for Windows (Version 23.0, IBM Japan Ltd., Tokyo, Japan).

## 3. Results

Among 1,515 patients with T2DM, the results of the subjects who had been followed for less than five years (*N* = 946) were considered to be inadequate for the assessment of longitudinal changes of renal function. We further excluded patients who were older than 65 years of age at baseline (*N* = 174) and/or those with incomplete data (*N* = 51); consequently 344 subjects (247 males and 97 females) were included in the analysis. The mean observation period of the 344 subjects in the present study was 8.1 ± 1.4 years (2,800 person-years of follow-up). The baseline characteristics of the study population are presented in Table 1. The prevalence of DR, SBP, and LDL-cholesterol values was significantly higher in females than in males. The mean annual change in eGFR was −3.5 ± 2.7%/year in females and −2.0 ± 2.2%/year in males (*P* < 0.001), and female sex was significantly associated with a larger decline of eGFR (B, −1.025; 95% CI, −1.645–−0.405). A greater number of females were “rapid decliners” compared to males (34.0% versus 17.4%, *P* = 0.001). The eGFR values at the endpoint were ≥60 mL/min/1.73 m^2^ in 285 subjects (209 males and 76 females), 30–59.9 mL/min/1.73 m^2^ in 46 subjects (32 males and 14 females), and <30 mL/min/1.73 m^2^ in 13 subjects (6 males and 7 females). All of patients with endpoint eGFR values of <30 mL/min/1.73 m^2^ were rapid decliners.

Table 2 presents the results of the univariate and multivariable linear regression analyses, separated by sex. In both sexes, the baseline DR and proteinuria were significantly associated with a larger decline of eGFR after multivariable adjustment. HbA1c and LDL-cholesterol were significantly associated with an annual eGFR decline in females only. Furthermore, interactive effects were observed between sex and the DR, proteinuria, HbA1c, or LDL-cholesterol levels on the annual eGFR decline (*P* < 0.05), indicating that females with these risk factors were at a greater risk of eGFR decline. The statistical significance of the associations of diabetes duration and SBP with renal function decline disappeared after the multivariable adjustment.  Table 3 shows the effects of the combination of sex and metabolic status abnormalities on annual eGFR decline. Females with poor HbA1c and/or LDL-cholesterol control showed a significantly greater annual eGFR decline than males with good control; however, this difference was not observed in females with good control. The combination of sex and SBP control did not have a statistically significant effect on the annual eGFR decline.  Table 4 shows the mean values for metabolic profiles during the observation period and the longitudinal influence of female sex on the mean values and the risks for abnormal metabolic profiles. The mean LDL-cholesterol level in females remained higher during the initial four years of the observation period. Although the mean HbA1c level in females did not differ markedly from that in males, the longitudinal association analyses showed that females had poorer control of their HbA1c and LDL-cholesterol levels compared to males during the observation period.

Table 5 shows the clinical characteristics of 41 females and 41 PS-matched males. The basal characteristics were not different between the males and the females, and their HbA1c, LDL-cholesterol, and SBP level were not well controlled. The females exhibited a greater annual eGFR decline than the males (−3.3 ± 2.5%/year versus −1.7 ± 1.6%/year, *P* = 0.001).

Lastly, bootstrap analyses were performed to verify the sex differences in eGFR decline. The results using 1,000 replicated datasets by the bootstrap approach further confirmed that females exhibited a larger annual eGFR decline than males in both unmatched (median B value, −1.025; 95% CI, −1.597–−0.457) and PS-matched populations (−3.3 ± 2.5%/year versus −1.7 ± 1.6%/year, *P* = 0.004).

## 4. Discussion

In the present study, females exhibited a significantly greater decline in renal function than males, and the results from the PS matching model supported this association. HbA1c and LDL-cholesterol were significantly associated with annual eGFR decline in females only. These preliminary findings suggest that the association between poor metabolic control and annual eGFR decline is more common in females. Moreover, this study is, to our knowledge, the first report to show that the association between retinopathy and rapid renal function decline is more pronounced in females than in males. It has been proposed that different mechanisms underlie the development of diabetic vascular complications in females and males [10, 25]. Previous studies have revealed stronger relationships between retinopathy and CVD [26] and between renal dysfunction and CVD [27] in females. Taken together, the diabetic vascular complications in females were considered to be highly related to each other and there may be female-specific risks and/or a vulnerability to vascular dysfunction underlying these relationships.

In this study, female sex was significantly associated with a larger decline of eGFR (B, −1.025; 95% CI, −1.645–−0.405), and rapid decliners were found more often in females (34.0%) than in males (17.4%). A recent meta-analysis, which included more than 200,000 individuals with type 1 diabetes, revealed that the pooled female-to-male ratio of the standardized mortality ratio for fatal renal disease was 1.44 (95% CI 1.02–2.05) [11]. Females with T2DM have also been reported to be at a higher risk of diabetic nephropathy and renal dysfunction in previous studies [16, 25, 28, 29], while other studies have indicated a higher risk in males [16, 30]. Although the reason for the inconsistent results regarding sex differences in the risk of renal dysfunction in T2DM patients is unclear, it may be due to disparities in the participants' background characteristics and/or the various definitions of renal dysfunction. de Hauteclocque et al. assessed rapid eGFR decline using longitudinal serum creatinine data (as we did in our present study) and showed that male gender was independently associated with a rapid decline of renal function [30]. In our study population, however, the baseline metabolic profile was obviously worse than in the population of this previous study [30] and was comparable to or slightly worse than those in the other studies that indicated a higher risk of renal dysfunction in females with diabetes [25, 28]. It should also be noted that we examined a relatively early stage of renal dysfunction in this study. This is shown by the fact that only 17.2% of subjects exhibited an eGFR of <60 mL/min/1.73 m^2^ at the endpoint. Thus far, it has been reported that females with T2DM have a higher risk of severe renal dysfunction (renal impairment defined as a decrease in Cockcroft-Gault estimated creatinine <60 mL/min or a doubling of plasma creatinine) [28] and advanced diabetic kidney disease (an eGFR <30 mL/min/1.73 m^2^) [29]. The present study provided new information suggesting that among the Japanese T2DM patients, in whom baseline metabolic profiles of most subjects could not be controlled, females were at a higher risk of early-stage renal dysfunction.

The females of the present study exhibited poorer metabolic statuses during the observation period, and baseline HbA1c and LDL-cholesterol levels were associated with annual eGFR decline in females only. In order to diminish the sex disparities in the baseline characteristics and to confirm whether the decline in renal function in females is greater than in males with similar baseline characteristics, we conducted a supplementary analysis using a PS matching model. In this supplementary analysis, where the baseline metabolic statuses of the males and females were equally poor, we confirmed that there was a greater annual eGFR decline in the female subjects. It is well known that females (especially younger females) are more insulin sensitive and are at lower risk of vascular disease than males [8, 14]. Estrogen has various vasoprotective effects (e.g., vasodilation, lipid profile improvement, antioxidation, anti-inflammation, and antifibronic effects) and plays an important role in this advantage of females [7, 31]. In the kidney, estrogen activates metalloproteinase enzymes and nitric oxide synthesis, inhibits the renin-angiotensin system and mesangial cell proliferation, and reduces inflammation [29]. In hyperglycemic states such as diabetes and prediabetes, however, these advantages of estrogen appear to be diminished or abolished [6, 8, 14, 32]. Females with T2DM have been reported to exhibit a reduced productivity of estrogen in comparison to healthy counterparts, even in their twenties [7, 33]. Furthermore, patients with diabetes appear to exhibit an impaired balance of the estrogen receptors (ER*α* and ER*β*). The increased expression or activation of ER*β* over ER*α* could induce a higher level of oxidative stress, a proinflammatory profile, and the increased formation of atherosclerotic plaque [5]. It is considered that biologically, females in hyperglycemic states tend to exhibit a greater arteriosclerotic risk profile and greater endothelial dysfunction [6, 8, 14, 32], which suggests that both may have contributed to the increased vulnerability to vascular dysfunction (such as diabetic nephropathy) that was observed in the females of this study.

The present study is associated with some limitations. First, the retrospective study design and small sample size are crucial limitations. To reduce the selection bias, we conducted a supplementary analysis using the PS matching model, a technique that has been used in other recent retrospective studies [24]. Although inexact or incomplete matching might have affected the results of this study, we matched male patients with 42.3% of the female patients, with a median standardized difference after matching of 0.08, which indicates a satisfactory matching. In addition, we verified our findings using the bootstrap approach to address the small sample size. These validation results also indicated a greater decline in renal function in females than in males. Second, in the present study, we used the Japanese eGFR-estimating equation, not the CKD Epidemiology Collaboration (CKD-EPI) equation. However, even when analyzing our data using the CKD-EPI equation for Japanese population [34], females still exhibited a significantly greater decline in renal function than males (−2.4 ± 2.8%/year versus −1.6 ± 1.7%/year, *P* = 0.009). The findings obtained using the CKD-EPI equation were generally comparable to those using the Japanese eGFR-estimating equation (Supplemental Tables  1–3; see Supplementary Material available online at http://dx.doi.org/10.1155/2016/4626382). Third, the patients of our study population, which were from a diabetes care center, basically had poor metabolic control at their first visit. The higher risk of rapid renal function decline in females appears to manifest in such situations due to their biological vulnerability. Thus, it should be taken into account that it remains unknown whether female sex is a risk factor for renal dysfunction in other populations, especially diabetic patients whose metabolic profiles are well controlled. Fourth, information regarding the menopause status of the patients was not available in the present study. However, if we are to assume that menopause occurs between the ages of 45 and 55, then the pre- and postmenopause age groups were not independently associated with rapid eGFR decline. Lastly, we could not assess the relationship between albuminuria and a rapid eGFR decline due to a lack of data. Therefore, further investigations in larger populations, which include longitudinal and quantitative data on albuminuria and serum creatinine levels and other laboratory/descriptive information, are needed to verify our findings.

## 5. Conclusions

In summary, this exploratory longitudinal study showed that females exhibited a greater decline in renal function than males. An increased susceptibility to poor metabolic control might contribute to a higher risk of renal dysfunction in females with T2DM. Although further investigations are needed to verify these preliminary findings, we provided useful information on sex differences in renal dysfunction in patients with T2DM. Our study highlights the importance of the aggressive therapeutic interventions to improve metabolic profiles at an early stage, especially in females.

## Supplementary Material

The findings obtained using the CKD-EPI equation, i.e. the relationships between baseline variables and the annual CKD/EPI-eGFR change in males and females (Supplemental Table 1); the effects of the combination of sex and metabolic status-abnormalities on the annual CKD/EPI-eGFR decline (Supplemental Table 2); and the clinical characteristics of males and PS-matched females (Supplemental Table 3).

## Figures and Tables

**Table 1:** The baseline characteristics of the overall study population and separated by sex.

	All subjects	Males	Females	*P* value
	(*N* = 344)	(*N* = 247)	(*N* = 97)	
Age (years)	50.8 ± 9.1	49.9 ± 9.5	53.2 ± 7.6	0.001
Age at diagnosis of diabetes (years)	45.6 ± 9.5	44.6 ± 9.7	48.0 ± 8.7	0.003
Diabetes duration (years)	5.3 ± 6.1	5.3 ± 6.3	5.2 ± 5.4	0.924
Diabetic retinopathy				
No retinopathy (%)	279 (81.1)	210 (85.0)	69 (71.1)	0.008
NPDR (%)	56 (16.3)	33 (13.4)	23 (23.7)	
PDR (%)	9 (2.6)	4 (1.6)	5 (5.2)	
HbA1c (%)	9.7 ± 2.6	9.7 ± 2.7	9.8 ± 2.4	0.688
HbA1c (mmol/mol)	83.0 ± 28.5	82.6 ± 29.3	84.0 ± 26.5	0.692
eGFR (mL/min/1.73 m^2^)	96.5 ± 24.0	95.1 ± 21.7	99.9 ± 28.9	0.140
Proteinuria (%)	98 (28.5)	72 (29.1)	26 (26.8)	0.693
SBP (mmHg)	139.5 ± 24.1	136.9 ± 22.6	146.2 ± 26.4	0.002
DBP (mmHg)	85.5 ± 13.6	85.0 ± 13.1	86.7 ± 14.7	0.279
Hypertension (%)	180 (52.3)	118 (47.8)	62 (63.9)	0.008
Total-cholesterol (mmol/L)	5.5 ± 1.1	5.4 ± 1.1	5.9 ± 1.0	<0.001
LDL-cholesterol (mmol/L)	3.3 ± 0.9	3.2 ± 0.9	3.6 ± 0.9	<0.001
HDL-cholesterol (mmol/L)	1.5 ± 0.4	1.4 ± 0.4	1.6 ± 0.4	<0.001
Triglycerides (mmol/L)	1.8 ± 1.7	2.0 ± 1.9	1.5 ± 0.8	0.002
Dyslipidemia (%)	278 (80.8)	190 (76.9)	88 (90.7)	0.004
BMI (kg/m^2^)	24.3 ± 4.3	24.1 ± 4.1	24.7 ± 4.7	0.227
Overweight (%)	134 (39.0)	89 (36.0)	45 (46.4)	0.086
Ever-smoker (%)	202 (58.7)	184 (74.5)	18 (18.6)	<0.001
Therapy components				
Hypoglycemic agents				
Oral hypoglycemic agents (%)	97 (28.2)	58 (23.5)	39 (40.2)	0.003
Insulin (%)	20 (5.8)	15 (6.1)	5 (5.2)	1.000
Antihypertensive agents				
ACE inhibitors or ARBs (%)	18 (5.3)	11 (4.5)	7 (7.4)	0.286
Others (%)	30 (8.7)	17 (6.9)	13 (13.4)	0.059
Hypolipidemic agents				
Statins (%)	22 (6.4)	6 (2.4)	16 (16.7)	<0.001
Fibrates (%)	12 (3.5)	4 (1.6)	8 (8.3)	0.005
Others (%)	4 (1.2)	3 (1.2)	1 (1.0)	1.000

Data are presented as means ± SD or number (%).

ACE: angiotensin-converting enzyme; ARB: angiotensin receptor blocker.

**Table 2:** The relationships between baseline variables and the annual eGFR change in males and females.

	Univariate analysis		Multivariable analysis	
	*β* ^a^	*P* value	*β* ^a^	*P* value
Males				
DR (yes/no)	**−0.264**	**<0.001**	**−0.161**	**0.019**
Proteinuria (yes/no)	**−0.243**	**<0.001**	**−0.139**	**0.041**
Diabetes duration (years)	**−0.158**	**0.017**	−0.097	0.142
HbA1c (% or mmol/mol)	**−0.135**	**0.036**	−0.084	0.182
SBP (mmHg)	**−0.136**	**0.032**	−0.065	0.301
LDL-cholesterol (mmol/L)	−0.006	0.920	—	—
Overweight (yes/no)	0.062	0.332	—	—
Ever-smoker (yes/no)	−0.021	0.745	—	—
Females				
DR (yes/no)	**−0.465**	**<0.001**	**−0.248**	**0.022**
Proteinuria (yes/no)	**−0.344**	**<0.001**	**−0.207**	**0.026**
Diabetes duration (years)	**−0.256**	**0.013**	−0.160	0.101
HbA1c (% or mmol/mol)	**−0.377**	**<0.001**	**−0.191**	**0.049**
SBP (mmHg)	**−0.243**	**0.018**	−0.127	0.154
LDL-cholesterol (mmol/L)	**−0.267**	**0.009**	**−0.182**	**0.041**
Overweight (yes/no)	0.058	0.587	—	—
Ever smoker (yes/no)	0.049	0.644	—	—

^a^Adjusted for baseline age.

**Table 3:** The effects of the combination of sex and metabolic status-abnormalities on the annual eGFR decline.

Sex	Clinical feature	*N*	Annual eGFR change (%/year)	*β* ^a^	*P* value
Male	HbA1c < 7.0% (53.0 mmol/mol)	43	−1.4 ± 1.9	0	—
	HbA1c ≥ 7.0% (53.0 mmol/mol)	204	−2.2 ± 2.2	−0.055	0.464
Female	HbA1c < 7.0% (53.0 mmol/mol)	13	−2.7 ± 1.4	−0.083	0.139
	HbA1c ≥ 7.0% (53.0 mmol/mol)	84	−3.6 ± 2.9	**−0.234**	**0.003**

Male	LDL-cholesterol < 3.4 mmol/L	146	−2.1 ± 2.3	0	—
	LDL-cholesterol ≥ 3.4 mmol/L	101	−2.0 ± 2.0	0.030	0.577
Female	LDL-cholesterol < 3.4 mmol/L	38	−3.1 ± 1.8	−0.090	0.108
	LDL-cholesterol ≥ 3.4 mmol/L	59	−3.8 ± 3.2	**−0.189**	**0.001**

Male	SBP < 140 mmHg	147	−1.8 ± 2.0	0	—
	SBP ≥ 140 mmHg	100	−2.4 ± 2.4	−0.019	0.561
Female	SBP < 140 mmHg	40	−2.8 ± 2.6	**−0.117**	**0.033**
	SBP ≥ 140 mmHg	57	−4.0 ± 2.7	**−0.207**	**<0.001**

Data are presented as means ± SD.

^a^Adjusted for baseline age, diabetes duration, DR, proteinuria, overweight, ever-smoker, HbA1c (except for first model), LDL-cholesterol (except for second model), and SBP (except for third model).

**Table 4:** Metabolic profiles during the observation period^a^.

	Baseline^b^	Year 1^b^	Year 2^b^	Year 3^b^	Year 4^b^	Year 5^b^	Clinical parameters			Abnormal level^c^		
							Β^e^	SE	*P* value	OR^e^	95% CI	*P* value
HbA1c (%)												
Males	9.7 ± 0.2	7.5 ± 0.1	7.5 ± 0.1	7.5 ± 0.1	7.7 ± 0.1	7.7 ± 0.1	0	—	—	1	—	—
Females	9.8 ± 0.3	7.6 ± 0.1	7.5 ± 0.1	7.6 ± 0.1	7.4 ± 0.1	7.4 ± 0.1^d^	0.25	0.14	0.084	**1.66**	**1.09–2.53**	**0.018**
HbA1c (mmol/mol)												
Males	82.6 ± 1.9	58.8 ± 1.3	59.1 ± 1.1	58.9 ± 1.1	60.3 ± 1.1	61.0 ± 1.1	0	—	—	1	—	—
Females	84.0 ± 2.7	60.1 ± 1.6	58.7 ± 1.5	59.4 ± 1.6	58.0 ± 1.3	57.3 ± 1.3^d^	2.65	1.55	0.087	**1.66**	**1.09–2.53**	**0.018**
LDL-cholesterol (mmol/L)												
Males	3.2 ± 0.1	3.0 ± 0.1	3.0 ± 0.1	3.0 ± 0.1	3.1 ± 0.1	3.1 ± 0.1	0	—	—	1	—	—
Females	3.6 ± 0.1^d^	3.4 ± 0.1^d^	3.5 ± 0.1^d^	3.5 ± 0.1^d^	3.3 ± 0.1^d^	3.2 ± 0.1	**0.31**	**0.08**	**<0.001**	**1.96**	**1.39–2.76**	**<0.001**
SBP (mmHg)												
Males	136.9 ± 1.4	133.6 ± 1.3	133.6 ± 1.1	134.1 ± 1.2	136.4 ± 1.2	135.9 ± 1.1	0	—	—	1	—	—
Females	146.2 ± 2.7^d^	136.6 ± 2.4	135.9 ± 1.9	137.1 ± 1.7	134.5 ± 2.0	135.0 ± 1.8	−2.00	1.62	0.218	0.82	0.58–1.16	0.252

^a^The longitudinal data were described only up to year 5 of the observation period, in which the sample size was sufficiently large to calculate the mean and SE values for the metabolic profiles.

^b^Data are presented as means ± SE.

^c^Abnormalities of HbA1c (≥7.0%, i.e. ≥53.0 mmol/mol), LDL-cholesterol (≥3.4 mmol/L), and SBP (≥140 mmHg).

^d^ *P* < 0.05 versus males analyzed by Student's *t*-test.

^e^Longitudinal analyses for the entire observational period (8.1 ± 1.4 years) were adjusted for age, diabetes duration, DR, proteinuria, overweight, ever-smoker, HbA1c (except for first and second model), LDL-cholesterol (except for third model), and SBP (except for fourth model).

**Table 5:** The clinical characteristics of males and PS-matched females.

	Males	Females	*P* value
	(*N* = 41)	(*N* = 41)	
Annual eGFR change (%/year)	**−1.7 ± 1.6**	**−3.3 ± 2.5**	**0.001**
Variables used in the PS matching			
Age (years)	52.9 ± 7.4	51.1 ± 9.0	0.318
Diabetes duration (years)	4.9 ± 4.9	4.4 ± 4.9	0.703
DR (yes/no)	2 (4.9)	6 (14.6)	0.264
HbA1c (%)	9.5 ± 2.7	9.4 ± 2.2	0.915
HbA1c (mmol/mol)	80.1 ± 29.8	79.5 ± 24.0	0.915
Proteinuria (yes/no)	6 (14.6)	10 (24.4)	0.404
SBP (mmHg)	141.2 ± 21.8	143.0 ± 22.9	0.716
LDL-cholesterol (mmol/L)	3.3 ± 0.8	3.3 ± 0.6	0.969

Data are presented as means ± SD or number (%).
