# Supplementary material for: Sex Differences in the Renal Function Decline of Patients with Type 2 Diabetes
Source: J Diabetes Res. 2016 May 9;2016:4626382. doi: 10.1155/2016/4626382 (PMC4876234; doi:10.1155/2016/4626382)
Supplement: Supplementary file 1 — The findings obtained using the CKD-EPI equation, i.e. the relationships between baseline variables and the annual CKD/EPI-eGFR change in males and females (Supplemental Table 1); the effects of the combination of sex and metabolic status-abnormalities on the annual CKD/EPI-eGFR decline (Supplemental Table 2); and the clinical characteristics of males and PS-matched females (Supplemental Table 3). [file 4626382.f1.pdf]

Supplemental Table 1. The relationships between baseline variables and the annual CKD/EPI-eGFR change in males and females

	Univariate analysis		Multivariable analysis	
	β[a]	*P*-value	β[a]	*P*-value
Males				
DR (yes/ no)	**-0.329**	**<0.001**	**-0.213**	**0.001**
Proteinuria (yes/ no)	**-0.300**	**<0.001**	**-0.179**	**0.007**
Diabetes duration (years)	**-0.179**	**0.007**	-0.098	0.125
HbA1c (% or mmol/mol)	**-0.154**	**0.016**	-0.089	0.145
SBP (mmHg)	**-0.143**	**0.024**	-0.052	0.397
LDL-cholesterol (mmol/L)	-0.014	0.823	-	-
Overweight (yes/ no)	0.032	0.618	-	-
Ever smoker (yes/ no)	-0.009	0.890	-	-
Females				
DR (yes/ no)	**-0.420**	**<0.001**	**-0.235**	**0.030**
Proteinuria (yes/ no)	**-0.433**	**<0.001**	**-0.322**	**0.001**
Diabetes duration (years)	**-0.216**	**0.036**	-0.147	0.130
HbA1c (% or mmol/mol)	**-0.314**	**0.002**	-0.117	0.222
SBP (mmHg)	-0.201	0.051	-0.054	0.540
LDL-cholesterol (mmol/L)	**-0.313**	**0.002**	**-0.209**	**0.019**
Overweight (yes/ no)	0.074	0.485	-	-
Ever smoker (yes/ no)	0.017	0.872	-	-

[a] Adjusted for baseline age.

Supplemental Table 2. The effects of the combination of sex and metabolic status-abnormalities on the annual CKD/EPI-eGFR decline

Sex	Clinical feature	N	Annual CKD/EPI-eGFR change (%/year)	β[a]	*P*-value
Male	HbA1c < 7.0% (53.0 mmol/mol)	43	-1.1 ± 1.0	0	-
	HbA1c ≥ 7.0% (53.0 mmol/mol)	204	-1.7 ± 1.8	-0.030	0.694
Female	HbA1c < 7.0% (53.0 mmol/mol)	13	-1.7 ± 1.0	-0.032	0.570
	HbA1c ≥ 7.0% (53.0 mmol/mol)	84	-2.5 ± 3.0	-0.115	0.139
Male	LDL-cholesterol < 3.4 mmol/L	146	-1.6 ± 1.7	0	-
	LDL-cholesterol ≥ 3.4 mmol/L	101	-1.6 ± 1.6	0.023	0.664
Female	LDL-cholesterol < 3.4 mmol/L	38	-1.8 ± 1.5	-0.006	0.917
	LDL-cholesterol ≥ 3.4 mmol/L	59	-2.8 ± 3.3	**-0.134**	**0.023**
Male	SBP < 140 mmHg	147	-1.4 ± 1.4	0	-
	SBP ≥ 140 mmHg	100	-1.9 ± 2.0	0.004	0.935
Female	SBP < 140 mmHg	40	-1.8 ± 2.5	-0.044	0.423
	SBP ≥ 140 mmHg	57	-2.8 ± 2.9	-0.101	0.096

Data are presented as means ± SD.

[a] Adjusted for baseline age, diabetes duration, DR, proteinuria, overweight, ever smoker, HbA1c (except for first model), LDL-cholesterol (except for second model) and SBP (except for third model).

Supplemental Table 3. The clinical characteristics of males and PS-matched females

	Males (N = 41)	Females (N = 41)	*P*-value
Annual CKD/EPI-eGFR change (%/year)	-1.4 ± 1.0	-2.1 ± 2.4	0.092
Variables used in the PS matching			
Age (years)	52.9 ± 7.4	51.1 ± 9.0	0.318
Diabetes duration (years)	4.9 ± 4.9	4.4 ± 4.9	0.703
DR (yes/ no)	2 (4.9)	6 (14.6)	0.264
HbA1c (%)	9.5 ± 2.7	9.4 ± 2.2	0.915
HbA1c (mmol/mol)	80.1 ± 29.8	79.5 ± 24.0	0.915
Proteinuria (yes/ no)	6 (14.6)	10 (24.4)	0.404
SBP (mmHg)	141.2 ± 21.8	143.0 ± 22.9	0.716
LDL-Cholesterol (mmol/L)	3.3 ± 0.8	3.3 ± 0.6	0.969

Data are presented as means ± SD or number (%).
